# Evaluating broad-scale system change using the Consolidated Framework for Implementation Research: challenges and strategies to overcome them

**DOI:** 10.1186/s13104-018-3650-9

**Published:** 2018-08-04

**Authors:** Jennifer N. Hill, Sara M. Locatelli, Barbara G. Bokhour, Gemmae M. Fix, Jeffrey Solomon, Nora Mueller, Sherri L. LaVela

**Affiliations:** 1 Center for Evaluation of Practices and Experiences of Patient-Centered Care (CEPEP), Center of Innovation for, Complex Chronic Health Care (CINCCH), Edward Hines Jr. VA Hospital, 5000 S. 5th Ave (151H), Hines, IL 60141 USA; 2 Center for Healthcare Organization and Implementation Research (CHOIR), ENRM Veterans Affairs Medical Center, Center for Evaluating Patient-Centered Care (EPCC), Boston University School of Public Health, Edith Nourse Rogers Memorial VA Hospital, 200 Springs Rd, Bedford, MA 01730 USA; 3 Center for Healthcare Organization and Implementation Research (CHOIR), VA Health Services Research and Development Service, Evaluating Patient-Centered Care (EPCC), Boston University School of Public Health, Edith Nourse Rogers Memorial VA Hospital, 200 Springs Rd, Bedford, MA 01730 USA; 4 Evaluating Patient-Centered Care (EPCC), Edith Nourse Rogers Memorial VA Hospital, 200 Springs Rd, Bedford, MA 01730 USA; 5 Center for Evaluation of Practices and Experiences of Patient-Centered Care (CEPEP), Center of Innovation for Complex Chronic Health Care (CINCCH), Center for Healthcare Studies, Institute for Public Health and Medicine General Internal Medicine and Geriatrics, Edward Hines Jr. VA Hospital, Northwestern University, Feinberg School of Medicine, 5000 S. 5th Ave (151H), Hines, IL 60141 USA

**Keywords:** Evaluation, Implementation science, Theory, Framework, Consolidate framework for implementation research

## Abstract

**Objective:**

The objective of this paper is to demonstrate the utility of the CFIR framework for evaluating broad-scale change by discussing the challenges to be addressed when planning the assessment of broad-scale change and the solutions developed by the evaluation team to address those challenges. The evaluation of implementation of Patient-centered Care and Cultural Transformation (PCC&CT) within the Department of Veterans Affairs (VA) will be used as a demonstrative example. Patient-Centered Care (PCC) is personalized health care that considers a patient’s circumstances and goals. The Department of Veterans Affairs (VA) is working towards implementing PCC throughout its healthcare system, comprised of multiple interventions with a singular long-term goal of cultural transformation, however little is known about the factors influencing its implementation. This paper discusses the issues that arose using CFIR to qualitatively assess the factors influencing implementation of cultural transformation.

**Results:**

Application of CFIR to this broad-scale evaluation revealed three strategies recommended for use in evaluating implementation of broad-scale change: (1) the need for adapted definitions for CFIR constructs (especially due to new application to broad-scale change), (2) the use of a mixed deductive-inductive approach with thematic coding to capture emergent themes not encompassed by CFIR, and (3) its use for expedited analysis and synthesis for rapid delivery of findings to operational partners. This paper is among the first to describe use of CFIR to guide the evaluation of a broad-scale transformation, as opposed to discrete interventions. The processes and strategies described in this paper provide a detailed example and structured approach that can be utilized and expanded upon by others evaluating implementation of broad-scale evaluations. Although CFIR was the framework selected for this evaluation, the strategies described in this paper including: use of adapted definitions, use of mixed deductive-inductive approach, and the approach for expedited analysis and synthesis can be transferred and tested with other frameworks.

## Background

Improving performance and initiating broad-scale change at the organizational level in healthcare often involves multiple interventions, or a collection of interventions including complex, multi-faceted interventions needing careful coordination and adaptation to the specific context in which there are being implemented [1]. While understanding the process of dissemination of these practices is a priority [2] and efforts have been made to identify and describe mechanisms for change at the health system level when implementing complex multi-dimensional interventions [1], challenges exist in evaluating implementation of these complex interventions.

In evaluation, theories and frameworks describe and prescribe aspects of an assessment, rooted in the needs or requirements of the customer and the purpose of the inquiry. These include activities or strategies, methods choices, and the responsibilities of and products to be provided by the evaluators [3]. Evaluators must consider the complexity and coordination of the multiple interventions when selecting an appropriate evaluation strategy, but often end up relying on evaluation of the interventions individually due to the paucity of frameworks available for evaluating broad-scale change requiring multiple interventions.

The Consolidated Framework for Implementation Research (CFIR) has primarily been used to evaluate implementation of single, discrete interventions or programs [4–6], yet it may also be particularly useful for evaluating broad-scale programs implemented by large, integrated healthcare systems. The CFIR complements interventions built on process-oriented theories focused on how implementation should be planned, organized, and scheduled [7], aspects which are critical when coordinating and evaluating implementation of multiple, complex interventions. CFIR offers a comprehensive, unifying taxonomy of constructs related to the intervention, inner and outer settings, characteristics of individuals, and implementation process [8]. Because CFIR offers a wide-reaching set of constructs, it is possible and practical to apply these constructs as a comprehensive set of a priori codes for deductive coding. This, in turn, provides a means to expedite the analysis of large amounts of qualitative data [9] and facilitates the rapid turnaround of recommendations to leadership, operations partners or programs.

The objective of this paper is to demonstrate the utility of the CFIR framework for evaluating broad-scale change by discussing the challenges to be addressed when assessing broad-scale change and the solutions developed to addressing those challenges using the evaluation of implementation of Patient-centered Care and Cultural Transformation (PCC&CT) within the Department of Veterans Affairs (VA) as a demonstrative example.

### PCC&CT in VA

The VA model of patient-centered care (PCC) focuses on whole person care, by providing care that is personalized, proactive, and patient-driven [10]. The VA’s commitment to PCC was solidified with the creation of the Office of Patient-Centered Care and Cultural Transformation (OPCC&CT) in 2010 [10] which was established to promote cultural transformation in VA. To achieve this task, OPCC&CT used an approach that included a broad range of interventions and innovations that required piloting and evaluating, as well as testing of many different strategies for supporting their implementation across the system at the clinical and organizational level. Examples of interventions or innovations include: redesigning the environment of care to make it more comfortable and inviting (e.g., use of more natural light, providing better maps and way-finding for navigating the hospital) and providing training and promoting use of diverse approaches by clinicians where the patient is recognized as the primary member of a supportive team with an equal voice and with the choice of goals that may encompass all facets of their life, even those beyond primary health concerns [10].

OPCC&CT sought to understand how PCC was being implemented, and engaged health services researchers to conduct an implementation evaluation; given the size and scope of the transformation, two groups were selected to conduct the evaluation (located in Chicago and Boston). The goal of the evaluation described in this manuscript, a component of a larger evaluation, was to develop a set of recommendations for future rollout of PCC to the broader VA organization by describing the key lessons learned from understanding individual and organizational factors, key barriers and facilitators, and the strategies used and their impact on implementation; the results of which have been expected for publication elsewhere [11].

## Main text

### Methods

#### Challenges in assessing implementation of PCC&CT

This study used a realist approach to evaluation of PCC in VA [12] which recognizes that changes resulting from implementation of interventions/programs occur in complex and dynamic ways and initiate result in both planned and unplanned processes and outcomes [12]. Given that the evaluation was utilizing CFIR in a new way, the evaluation team utilized the following approach for planning for its use in the evaluation: (1) assessing its fit for the application, (2) closely tying the methodological approach and analytic strategy to the framework, and (3) projecting the use of the framework to structure a set of recommendations that could be used by the operations partner to support enhancement of implementation.

#### Identifying a framework and assessing its fit

Prior to the start of the evaluation, discussions with leadership in OPCC&CT focused on the goals of the program office to: (1) describe ongoing implementation efforts of the multiple interventions across the Centers of Innovation (COIs) and (2) to understand the factors influencing those implementation efforts. Recognizing the complexity of the evaluation, the team selected CFIR to plan and guide the implementation evaluation because of the comprehensive nature of its constructs and the flexibility offered in recommendations of its use [12]. Also, the comprehensive nature of the framework lends itself to use as an initial structure coding [13] because the dynamic and numerous constructs offer coverage for wide-ranging themes and ensures the capture of those factors important to implementation [14].

The evaluation team used the “menu of constructs” process which involves identifying and including only constructs essential to the evaluation, which facilitates shorter, focused interviews and expedited analysis [12]. The evaluation teams had exploratory discussions with PCC leaders to gain a preliminary understanding of ongoing and planned innovations and their questions and goals for the evaluation. Following these discussions, the two evaluation teams met to review the scope of the evaluation and the constructs of CFIR that were most relevant and applicable to the evaluation [12] and critical to the questions and goals of key leadership, and followed that with a discussion with OPCC&CT to ensure the “essential constructs” needed to meet the evaluation goals were included. Specific reasons for the selection of each of the constructs can be found in Table 1. As recommended in a recent systematic review of use of CFIR, the framework was integrated into the evaluation design, data collection, and analysis [15] (Table 1).

**Table 1:** CFIR constructs and sample interview questions

Construct	Rationale for Selection	Sample questions
Domain: intervention characteristics		
Intervention source	[Evaluation team] The intervention was ongoing, evaluation team wanted to capture historical information	Can you tell me a bit about the history of transforming the organization to patient-centered care?
Evidence strength and quality	[Evaluation team] Collect information on perceptions of evidence regarding PCC	What clinical or research evidence, or literature were you aware of that supported use of an intervention like this?
Relative advantage	[Evaluation team] Advantages and goals of transformation and expectations of practice	What is your understanding of the goals of this initiative? How is this different from what you were doing?
Adaptability	[Evaluation team] Document adaptations, especially given the broad scope of the transformation	What were challenges you encountered? What adaptations were made to overcome these challenges?
Complexity	[Evaluation team] Gather information on perceived complexity of the PCC cultural transformation	How difficult would you say it has been to implement the intervention?
Cost	[Evaluation team] Funding available from OPCC&CT and money contributed by facility	What types of funding have you received since becoming a COI to implement PCC innovation(s)? Did your facility incur any additional costs not covered by the funds?
Domain: outer setting		
Patient needs and resources	[OPCC&CT and evaluation team] Level of patient involvement in the transformation	How are interventions selected? How do patients become engaged? What feedback is collected from them?
Domain: inner setting		
Implementation climate\culture	[OPCC&CT and evaluation team] Receptivity to the transformation	What was your perception of staff attitudes about the patient-centered care changes?
Tension for change	[Evaluation team] Is/was there a perceived need for change present?	What was your perception of the need for change?
Relative priority	[OPCC&CT and evaluation team] Level of priority of transformation in the real-world, clinical setting	Compared to other demands in the organization, how much of a priority is PCC?
Organizational incentives\rewards	[Evaluation team] Feedback received from OPCC&CT and individual/facility-level	How is feedback provided to staff on progress toward goals?
Goals and feedback	[OPCC&CT and evaluation team] Process for selecting and setting facility goals	How are goals communicated to staff? How is progress evaluated?
Leadership engagement	[OPCC&CT and evaluation team] Level of leadership involvement and activities contributing to transformation	What level of involvement did leadership have with this initiative? What did they do?
Available resources	[OPCC&CT and evaluation team] Resources provided by OPCC&CT and those provided by facility leadership	To what extent are there additional resources available to support these efforts?
Domain: characteristics of the individual		
Knowledge and beliefs	[OPCC&CT and evaluation team] Impact of messaging about PCC on how it’s defined at the individual-level	What do you think about when you hear the term patient-centered care? What are its key aspects?
Domain: process		
Planning	[OPCC&CT and evaluation team] Identify process and selection of assembly of local implementation team	Once the decision was made to start this initiative, who was involved in planning?
Engaging: staff	[OPCC&CT and evaluation team] How and why staff are engaged in the PCC cultural transformation	How were staff members engaged to participate in the initiative? Training provided?
Engaging: champions	[OPCC&CT and evaluation team] If/how champions emerged—who, what, why	Who was involved in planning? Was there a particular person who led the charge? How does this person’s energy for this initiative affect ongoing efforts?
Executing	[OPCC&CT and evaluation team] Identify spread/touch of PCC to patients	How do you document that a patient has been recommended\ participated in a PCC innovation?
Reflecting and evaluating	[OPCC&CT and evaluation team] Site-level processes for tracking transformation progress	What is being done to evaluate implementation of the intervention? Patient outcomes?

#### Developing a methodological approach and analytic strategy

Qualitative data were collected through semi-structured interviews with key stakeholders at each VA facility. CFIR was used to guide development of the interview questions, as well as the coding structure and data analysis. Operational definitions were developed for the CFIR constructs selected for the evaluation (Table 2) based on consensus between the evaluation teams to ensure members collected and analyzed data based on the same understanding of the domains and constructs [16]. These definitions were used by the evaluation teams to inform development of an interview guide to be used by both groups (Table 2). The interviews were conducted by a team of experienced qualitative researchers. Interviews were audio-recorded and transcribed and descriptive field notes [17] were taken in instances where audio-recordings were not collected.

**Table 2:** Full list of CFIR constructs, general definitions and adapted definitions

CFIR construct	General definition	Adapted definitions
Domain: intervention characteristics		
Intervention source	Perception of key stakeholders about whether the intervention is externally or internally developed	History of PCC-related program(s) or practice(s) and perceived source of the initiative
Evidence strength and quality	Stakeholders’ perceptions of the quality and validity of evidence supporting the belief that the intervention will have desired outcomes	Perception of intervention patient-centeredness
Relative advantage	Stakeholders’ perception of the advantage of implementing the intervention versus an alternative solution	Perception of the advantage of intervention relative to current practices
Adaptability	The degree to which an intervention can be adapted, tailored, refined, or reinvented to meet local needs	How intervention was adapted to current setting
Trialability	The ability to test the intervention on a small scale in the organization, and to be able to reverse course (undo implementation) if warranted	The ability to test the intervention (pilot) and to de-implement if necessary
Complexity	Perceived difficulty of implementation, reflected by duration, scope, radicalness, disruptiveness, centrality, and intricacy and number of steps required to implement	How hard has this been to do (scope, radicalness, disruptiveness)
Design quality and packaging	Perceived excellence in how the intervention is bundled, presented, and assembled	How the intervention/initiative is presented to staff
Cost	Costs of the intervention and costs associated with implementing the intervention including investment, supply, and opportunity costs	Funds received, facility funds used, training and staff time
Domain: outer setting		
Patient needs and resources	The extent to which patient needs, as well as barriers and facilitators to meet those needs, are accurately known and prioritized by the organization	Identified patient needs, processes used to identify them, barriers and facilitators associated with meeting needs and strategies for engaging patients to identify ways to address them
Cosmopolitanism	The degree to which an organization is networked with other external organizations	Connections with non-VA organizations, &/or current ideas in the literature
Peer pressure	Mimetic or competitive pressure to implement an intervention; typically because most or other key peer or competing organizations have already implemented or are in a bid for a competitive edge	Relationship with other VAs/OPCC/other COIs
External policies and incentives	A broad construct that includes external strategies to spread interventions, including policy and regulations (governmental or other central entity), external mandates, recommendations and guidelines, pay-for-performance, collaboratives, and public or benchmark reporting	Details about policy, recommendations, guidelines, could be VA/OPCC
Domain: inner setting		
Structural characteristics	The social architecture, age, maturity, and size of an organization	Size and age of facility, maturity, existing environmental/structural characteristics
Networks and communications	The nature and quality of webs of social networks and the nature and quality of formal and informal communications within an organization	Informal and formal communication structures (i.e. SharePoint, newsletters, etc.)
Culture	Norms, values, and basic assumptions of a given organization	Norms and values of organization
Implementation climate	The absorptive capacity for change, shared receptivity of involved individuals to an intervention, and the extent to which use of that intervention will be rewarded, supported, and expected within their organization	Capacity of change, receptivity to PCC or intervention/innovation
Tension for change	The degree to which stakeholders perceive the current situation as intolerable or needing change	Perceived need for change
Compatibility	The degree of tangible fit between meaning and values attached to the intervention by involved individuals, how those align with individuals’ own norms, values, and perceived risks and needs, and how the intervention fits with existing workflows and systems	Perception of compatibility with existing norms, values, and practices
Relative priority	Individuals’ shared perception of the importance of the implementation within the organization	Perception of the importance of the change relative to existing priorities
Organizational incentives and rewards	Extrinsic incentives such as goal-sharing awards, performance reviews, promotions, and raises in salary, and less tangible incentives such as increased stature or respect	Goal sharing awards, performance measure, and other motivators
Goals and feedback	The degree to which goals are clearly communicated, acted upon, and fed back to staff, and alignment of that feedback with goals	Goals are communicated, enacted, evaluated, and fed back to staff
Learning climate	A climate in which: (a) leaders express their own fallibility and need for team members’ assistance and input; (b) team members feel that they are essential, valued, and knowledgeable partners in the change process; (c) individuals feel psychologically safe to try new methods; and (d) there is sufficient time and space for reflective thinking and evaluation	Leaders addressing the need for feedback of team members, staff involvement in defining and refining intervention/innovation, its goals, and evaluation of progress
Readiness for implementation	Tangible and immediate indicators of organizational commitment to its decision to implement an intervention	Degree to which the organization is ready to implement intervention/innovation
Leadership engagement	Commitment, involvement, and accountability of leaders and managers with the implementation	Commitment, involvement, and accountability of leaders and managers
Available resources	The level of resources dedicated for implementation and on-going operations, including money, training, education, physical space, and time	Money, training, education, physical space, and time
Access to knowledge and information	Ease of access to digestible information and knowledge about the intervention and how to incorporate it into work tasks	Availability and usefulness of intervention information
Domain: characteristics of the individual		
Knowledge and beliefs about the intervention	Individuals’ attitudes toward and value placed on the intervention as well as familiarity with facts, truths, and principles related to the intervention	Individuals’ attitudes toward and value placed on the intervention as well as familiarity with facts, truths, and principles related to the intervention
Self-efficacy	Individual belief in their own capabilities to execute courses of action to achieve implementation goals	Individual belief in capabilities to achieve implementation goals
Individual stage of change	Characterization of the phase an individual is in, as he or she progresses toward skilled, enthusiastic, and sustained use of the intervention	Changes in the individual’s disposition toward the change
Identification with organization	A broad construct related to how individuals perceive the organization, and their relationship and degree of commitment with that organization	Perception of the organization, and relationship and commitment to it
Other personal attributes	A broad construct to include other personal traits such as tolerance of ambiguity, intellectual ability, motivation, values, competence, capacity, and learning style	Perception of personal traits (i.e. motivation, values, capacity, and learning style)
Domain: process		
Planning	The degree to which a scheme or method of behavior and tasks for implementing an intervention are developed in advance, and the quality of those schemes or methods	Methods and tasks and for implementing an intervention identified
Engaging	Attracting and involving appropriate individuals in the implementation and use of the intervention through a combined strategy of social marketing, education, role modeling, training, and other similar activities	Presence and description of people (opinion leaders, implementation leaders, champions) who educated/trained, role modeled, etc)
Opinion leaders	Individuals in an organization who have formal or informal influence on the attitudes and beliefs of their colleagues with respect to implementing the intervention	Individual with informal or formal influence on the attitudes\beliefs of colleagues
Formally-appointed implementation leaders	Individuals from within the organization who have been formally appointed with responsibility for implementing an intervention as coordinator, project manager, team leader, or other similar role	Formally appointed leaders with clearly defined responsibilities related to implementation
Champions	Overcoming indifference or resistance that the intervention may provoke in an organization	Individuals who are formally appointed or informally volunteer to support change
External change agents	Individuals who are affiliated with an outside entity who formally influence or facilitate intervention decisions in a desirable direction	Outside groups who assist in intervention
Executing	Carrying out or accomplishing the implementation according to plan	Carrying out or accomplishing the implementation according to planned
Reflecting and evaluating	Quantitative and qualitative feedback about the progress and quality of implementation accompanied with regular personal and team debriefing about progress and experience	Steps to evaluate implementation and patient outcomes

A mixed deductive-inductive [18–20] approach to coding was used to analyze data from the interviews. In this approach, operational examples are used to define each construct and are used to create an initial code list to be used for the analysis [21]. Deductive coding was guided by CFIR [4] using a structured analytical tool (Table 1) to facilitate rapid qualitative analysis. Inductive coding was used to capture themes that were not represented in CFIR as a way to ensure coding was reflective of the data, especially given that CFIR had mainly been used to assess discrete interventions rather than larger scoping programmatic evaluations. Although inter-coder reliability was not calculated initially, the study-specific operational definitions and newly created inductive codes resulted in a high-level of agreement between coding teams requiring few consensus discussions, in cases where consensus discussions were required, there was 100% agreement after consensus (no discussions ended in disagreements or required a third coder).

#### Projecting use of CFIR for development of a structured set of recommendations

The final step of this process required synthesizing early findings of the evaluation for rapid use by our operations partner in the field. This process involved: (1) conducting an additional level of analyses on data within these key domains to define how and why the domain was salient in the context of the evaluation and (2) developing a set of recommendations that could be utilized by leadership to enhance implementation of the program. The evaluation team planned to utilize a similar process as the qualitative coding described above. Key domains were split between the evaluation teams and were independently analyzed by investigators to develop definitions which were then refined within each team. Next, a set of recommendations was developed by the individual evaluation teams assessing the key domains. Finally, several meetings were conducted with the full evaluation team to discuss the definitions of the key domains and the recommendations for finalization.

### Results

#### Strategies identified for using CFIR to evaluate implementation of broad-scale change

During the evaluation, the strategies identified by the evaluation team for use of the CFIR in this new application were tracked as they were considered essential components for understanding the approach to and use of CFIR for this broad scale evaluation. Aspects of each of these strategies were tracked through documents that were also used for completing required elements of the evaluation (such as meeting minutes). The strategies identified by the team and the documented aspects of those strategies are presented in the results below.

Three overarching strategies were identified during the evaluation including: (1) the creation of *adapted* definitions for the CFIR constructs to account for its application to the broad-scale evaluation (Table 1), (2) the use of a mixed deductive-inductive coding process which demonstrated the flexibility of use of the CFIR framework for complex evaluation in the *emergence* of both additional CFIR constructs not initially accounted for by the study team (Table 3) and several new key themes from the co-occurring inductive thematic coding (Table 4). Finally, (3) use of CFIR for the *rapid* analysis and synthesis of the data into key domains impacting implementation of PCC&CT to develop recommendations for the VA OPCC&CT leadership to support enhancement of implementation and expansion opportunities of the program (Table 5). These findings are described in further detail below.

**Table 3:** Planned and Emerging Constructs in Interviews from the CFIR

5 CFIR domains	Pre-selected constructs by OPCC&CT and evaluation team with direct interview questions	Additional constructs emerging from interviews
I. Intervention characteristics	Intervention source Evidence strength and quality Relative advantage Adaptability Complexity Cost	None
II. Outer setting	Patient needs	Cosmopolitanism Peer pressure External policies and incentives
III. Inner setting	Culture or implementation climate Tension for change Relative priority Organizational rewards and incentives Goals and feedback Leadership engagement Available resources	Structural Characteristics Networks and communications Compatibility Learning climate Access to knowledge and information
IV. Characteristics of individuals	Knowledge and beliefs of the intervention	Self-efficacy Individual stage of change Individual identification with organization Other personal attributes
V. Process	Planning Engaging Champions Executing Reflecting and evaluating	Opinion leaders Formally appointed internal implementation leaders External change agents

**Table 4:** Grounded thematic coding (inductive) thematic codes and definitions

Construct/theme	Rationale for selection	Definition and/or examples of themes
Role in VA	Evaluation team Understand implications of dual roles related to implementation	Official VA title and general roles and responsibilities outside of PCC (if applicable)
Difficulty implementing PCC	OPCC&CT and evaluation team Self-rated outcomes of PCC for use as a proxy measure of implementation success	A rating of the difficulties implementing PCC on a 1-10 scale, 10 being most difficult
Progress of PCC implementation		A rating of the progress implementing PCC, 10 being the best
PCC barriers	OPCC&CT and evaluation team Identification of factors influencing implementation for ongoing reporting and dissemination to the field	Barriers related to implementing PCC (dual coded with other CFIR/OTM constructs)
PCC facilitators		Facilitators related to PCC (dual coded with other CFIR/OTM constructs)
Creation story	Evaluation team Provide context for the transformation given the delayed start of the evaluation	Refers to the participant’s telling of the history of PCC at the site
Future plans	OPCC&CT and evaluation team Document innovative thinking occurring at the facility or unit level in relation to future planning/sustainability for consideration at national-level	The participants tells about future plans in regards to PCC initiatives
Golden nugget	Evaluation team Tags exemplary quotes representing any construct	Anything the analysis team thought especially salient; paired with other codes; used to identify key quotes exemplifying paired codes
“Halls and Walls”	OPCC&CT and evaluation team Renovations occurring or needed changes to the physical environment	Any mention of physical renovations; focus of OPCC
Key strategies	OPCC&CT and evaluation team Innovative or best practices for ongoing reporting and dissemination to the field	Key strategies for implementing PCC (i.e. What’s worked well in implementing PCC)

**Table 5:** Most salient constructs, description of data collected, recommendations developed, and operational tactics

Constructs	Brief description of data	Recommendation from evaluation team	Operational tactics
Patient needs and resources	Involvement of patients and their families was viewed as a critical component	Engagement of patients can occur through Informal and formal feedback mechanisms (such as surveys, interviews, or focus groups and consumer panels or counsels) Providing information to patients and families about new programs/treatments	Capitalize on formal feedback sessions for Veterans engagement Establish informal mechanisms to engage Veterans
Leadership engagement	Active and visible support from all levels of leadership facilitated implementation, and encouraged staff buy-in	Leadership engagement can occur through Actively and openly supporting PCC Supporting/encouraging staff involvement Seeking and acting upon staff feedback Identify leaders to be dedicated to PCC	Identify a core cadre of leaders who will be responsible for the cultural transformation
Structural characteristics	Organizational structures, processes, rules, were significant barriers (i.e. role clarity, misaligned performance measures)	Organizational structure and barriers can be addressed by Clarify goals of the program, expectations for staff Aligning performance measures with program goals and expectations of staff	Provide examples of how you have fostered flexibility to support PCC Identify areas where you built on synergies with other priority initiatives
Knowledge and beliefs of the intervention	Staff reported challenges incorporating PCC into their regular practice, often not understating their role in PCC	Clarifying roles and priorities of PCC for staff can be achieved by Providing examples of how PCC should be integrated into daily Ensuring that staff are accountable for incorporating PCC into practice	Provide clear examples of embodying PCC Adapt performance measures for PCC to align staff work with institutional priorities
Engaging staff	Providing opportunities for staff to engage and providing feedback on efforts were essential for staff buy-in	Enculturating staff can be achieved by Targeted training supporting expectations that all staff should embody the principles and incorporate them into every day Infuse PCC messages everywhere	Showcase support for creative and appropriate risk-taking in development of new PCC approaches among frontline staff
Champions and/or formally appointed champions	Naturally emerging champions and use of mid-level champions facilitated innovations derived from senior leadership	Fostering innovations and new ideas generated by staff can be achieved by Supporting naturally emerging champions and/or establishing mid-level champions Developing strategies to support innovations with feasible implementation	Identify middle managers as clinical champions to foster implementation of senior management-initiated innovation

#### Adapted definitions

As a first step, the evaluation team reviewed the CFIR domains and constructs and developed adapted definitions based on the study context, including: (1) the broad scope of the intervention(s), (2) the broad-scale change targeted, (3) the input and existing knowledge of the operations partner OPCC&CT, and (4) the goals of the evaluation including assessing what had already occurred and what was currently in progress. Some definitions required more adaptation than others. For example, the domain/construct “Intervention Characteristics/Complexity” or “Inner Setting/Culture” did not necessarily require adaptation of the definition, however the questions associated with measuring those constructs did have to be broader than those typically associated with a single-intervention evaluation. Adapted definitions for the other constructs are available in Table 2; and the standard short descriptions of these constructs can be found at the CFIR Wikipage [22].

Other domain constructs required adaptation of the definitions to fit the goals of the evaluation and the needs of the operations partner. For example, the domain/construct “Intervention Characteristics/Intervention Source” required adaptation from the standard short description “perceptions of key stakeholders about whether the intervention is externally or internally developed” [22] to the adapted definition “History of PCC-related program(s) or practice(s) and perceived source of the initiative.” The primary purpose for this adaptation was two-fold, (1) the participants interviewed in this study had some level of involvement in the implementation of PCC at their facility, and therefore were familiar with OPCC&CT and the source of the intervention and (2) OPCC&CT spent much time and energy on strategies to expose individuals to the PCC cultural transformation and knowledge of the source of the intervention was (presumably) widely known. Instead, the emphasis was placed on understanding the history of PCC at the facilities which, in some cases was the source of the intervention in that it was an innovation already present at a facility and adopted by OPCC&CT as recommendations for other facilities.

In another example, the domain/construct “Outer Setting/Patient Needs & Resources” was adapted from the standard short definition “the extent to which patient needs, as well as barriers and facilitators to meet those needs, are accurately known and prioritized by the organization” to the adapted definition “Identified patient needs, processes used to identify them, barriers and facilitators associated with meeting needs and strategies for engaging patients to identify ways to address them.” The need to adapt the definition for this construct was largely driven by the characteristics of the transformation, namely, its patient-centered and patient-driven nature. OPCC&CT was interested in more information beyond just understanding patient needs and the barriers/facilitators to addressing them, such as the processes and strategies for engaging patients as partners to identify, strategize, and address those needs.

#### Emergence of CFIR constructs (deductive) and new thematic codes (inductive)

CFIR is composed of 39 constructs, of which 19 were selected by the evaluation team to gather data on via target interview questions (Table 1); these constructs were selected as part of the “menu of constructs” approach [12] focusing on the essential questions to the evaluation.

Interview data revealed that out of the selected constructs targeted in the interview guide, all 19 (100%) were identified as important influences on the implementation of PCC. Several interview questions encouraged longer narrative type answers such as: “*What do you think about when you hear the term patient*-*centered care? What are the key elements for care to be patient*-*centered from your perspective?”* and *“Tell me a little bit about the history of transforming the organization to become more patient*-*centered.”* These types of questions, along with follow-up and probe questions which were asked to further explore participant’s perspectives resulted in the emergence of additional CFIR constructs beyond those selected in the menu of constructs process prior to the start of the evaluation. In fact, another 16 CFIR constructs emerged across 4 of the 5 CFIR domains (Table 3) when using deductive coding with the CFIR structured analytical tool (Table 2). Interestingly, although OPCC&CT and the evaluation team placed lesser emphasis on the factors in the outer setting and characteristics of individual domains and constructs, multiple additional constructs emerged in these two domains.

The mixed deductive-inductive approach to coding enabled the team to utilized thematic coding (inductive) to create codes for additional themes that: (1) were not fully represented by a CFIR construct, (2) provided context-specific details, or (3) offered advantages for organization of ideas. An example for each of these is provided below, and the thematic codes and their definitions are provided in Table 4.

For example, one of the emerging codes that was not fully represented by a CFIR construct was ‘key strategies.’ This code was used to explore key strategies utilized in implementation; an example that emerged from the data included taking chances with novel ideas that resulted in “*quick wins*” and “*sparks*” of innovation across the hospital that encouraged staff to embrace the idea of PCC. In another example, the code of ‘role in VA’ as a *context*-*specific detail* was used to differentiate the dual roles that some served (one OPCC-specific role and one in VA in general). For example, an individual might serve as a Patient-Centered Care Coordinator within the transformation and as a Nurse within a clinical role in an overall VA role, often referred to as “collateral duty”; a dual role that that could result in a dual perspective that should be differentiated.

Another code ‘creation story’ was used to capture the previous history of PCC efforts at the facility. This code was important; particularly given the fact that (1) the sites were selected as COIs, in part, based on their status as leaders in cultural transformation and (2) that the evaluation was being conducted after the transformation had already begun. Unlike the ‘Tension for Change’ construct within CFIR which is focused on identifying a ‘need’ for change (often reflective of a discrete issue or set of issues), the code “creation story” encourages a more narrative reflection on the setting in which PCC was being implemented at its inception.

Some codes simply offered advantages for organizing ideas such as the “PCC Barriers” and “PCC Facilitators” codes where all mentions of barriers and facilitators could be placed for easy access rather than having to search within CFIR construct codes to identify them. By examining where barriers and facilitators were double-coded with CFIR constructs, the team was able to determine overarching themes that hindered or facilitated implementation of PCC innovations. Similarly, the code “Golden Nugget” was utilized as a place to identify codes that stood out to the coding teams or that were emblematic or particularly successful or salient in regards to the construct and was used for easy identification of these exemplary quotes.

#### Rapid, actionable feedback

Finally, the initial discussions where key evaluation questions were identified by the PCC leadership and the evaluation team and were connected to the CFIR framework and study-specific definitions developed facilitated delivery of rapid, actionable feedback on the evaluation. The availability of these context-specific definition for the constructs allowed for identification of factors influencing implementation in an organized and easily accessible way. It also enabled the evaluation team to deliver a methodologically sound, prompt analysis of the data which facilitated development of timely, meaningful recommendations to the operational partner. To demonstrate this point, 107 interviews were conducted, transcribed, and analyzed over a period of approximately 5 months. The evaluation team used this assessment to create a set of recommendations that could be used to facilitate the development of strategies and processes to support future implementation efforts which was delivered at the end of the 6th month. These findings are explored further in Table 5.

These examples and others were reported as part of a white paper developed by the evaluation teams, which described in the OPCC&CT annual report as informing the strategies the office was taking in moving the program forward.

### Discussion

The multiple, complex interventions often required for implementing broad-scale change create challenges for evaluation teams. In particular, there are no guidelines or recommended frameworks for evaluating implementation of broad-scale change. This study is among a small number of studies to use CFIR for conceptualizing an evaluation and for guiding data collection, coding, and analysis [12] and one of the first to use it to evaluate a broad-scale system change. In addition, examples in which CFIR has been used to assess implementation involving multiple interventions aimed at broad-scale change are limited; the authors identified one other example in which CFIR was used to assess implementation of a continuum of psychosocial interventions [23]. As such, it required a number of steps and processes that exercised the flexibility of the framework in new ways. This paper describes the steps taken to plan an evaluation and the strategies developed to utilize the CFIR framework for evaluation of broad-scale change.

The appropriateness of the application of CFIR in the evaluation of this broad-scale change is demonstrated by the ability of the framework’s constructs to “fit” the data. This is evident by the fact that constructs that were not ‘pre-selected’ by the study team as potentially relevant to this large-scale implementation emerged from the data and were captured by the evaluation team post hoc [14]. The current study differs from other studies using CFIR to evaluate discrete interventions [4–6, 12] in which findings are nearly exclusively tied to the framework [24] which may not be appropriate for the evaluation of a broad-scale change.

The application of CFIR in the context of broad-scale change required the creation of adapted definitions to account for this unique application that was used both to develop interview questions and to analyze interview data. In another study of “complex system interventions” Smith et al. [25] described a number of adaptations including changing the names of domains and constructs within CFIR to address distinctive features of the interventions being studied as well as modifying definitions of the constructs to incorporate terminology and exemplary examples of the specific interventions. In this study, an evaluation was not conducted, rather CFIR was used to inform the development of new frameworks to be used in future evaluation efforts in process redesign (PR), patient-centered medical homes (PCMH), and care transitions. The work completed by this group is important because it not exposes the advantages of adapting and refining existing CFIR constructs and definitions, but also details the process of doing so. The current study builds upon this work not only by demonstrating adaptation of the CFIR constructs in the context of a broad scale evaluation, but also using those adapted definitions to design data collection tools and a supporting analytic framework.

Utilizing a mixed deductive-inductive approach [18, 19] allowed for the identification of themes that emerged that were not represented in CFIR that may be unique to evaluating large scale transformations, rather than discrete intervention implementation. These themes needed independent codes, offered context-specific information, or were grouped together for better organization. Further, the approach used in this evaluation builds upon the work of Damschroder and colleagues who used CFIR to evaluate a large-scale weight management program in VA but also shared some details about their process including choosing not to do parallel inductive coding, but remaining open to new themes (though the group felt that significant themes were encompassed by CFIR) [12]. One of the reasons the PCC evaluation team chose CFIR as a framework was its flexibility and the openness of the creators of the framework to test its flexibility and applicability. The current study suggests that while application of CFIR as a deductive analytical framework without inductive coding to allow for emergent themes is appropriate in some cases, that in other cases, utilizing inductive coding to capture those themes is vitally important.

The use of CFIR facilitated the rapid analysis and synthesis of a larger number of interviews in a short period of time, 107 interviews in 5 months, with final synthesis and delivery of findings by the end of month six. This evaluation approach resulted in a methodologically sound, easily digestible, and actionable set of findings and recommendations for the operations partners in a white paper entitled *Lessons from the Field for Implementing Patient*-*Centered Care and Cultural Transformation*. This proved critical for OPCC&CT as they quickly operationalized the findings and disseminated a document to the field and their stakeholders entitled: *Lessons from the Field*—*Operational Tactics for Implementing Patient Centered Care and Cultural Transformation* which proposed “operational tactics” or steps to addressing findings from the white paper described in OPCC&CTs Annual Report [26].

### Conclusions

Utilizing CFIR in a relatively new application, a broad scale evaluation with multiple interventions, yielded the identification of a number of important processes and insights that should be considered to expand its applications to future broad-scale evaluations. This study demonstrates the utility and value of utilizing a comprehensive framework with a directed, yet flexible approach to evaluation which has implications for the broader field of implementation science. A collection of programs with multiple interventions with sometimes staggered, sometimes simultaneous beginnings presents a very challenging, complex evaluation that requires a balance between focus and flexibility. The insights that emerged from the study suggest that application of frameworks to organize findings and ideas from these complex evaluation environments are critical to delivering well-formulated recommendations that are derived from data driven by a sound theoretical basis. This study not only provides continued contribution to the larger implementation literature about the fit of the constructs from frameworks themselves in action, but also the utility and practicality of use of these frameworks for different applications.

In addition, the key analytic processes described in this paper provide a detailed example and structured approach that can be utilized and expanded upon by others in the implementation science community conducting broad-scale evaluations. Although CFIR was the framework selected for this evaluation, the analytical processes described in this paper including: use of adapted definitions, value of using mixed deductive-inductive approach, and the approach for expedited analysis and synthesis can be transferred and tested with other frameworks. Continuing to test frameworks, in general, and reporting experiences with use of these frameworks in new ways provides continued important insight to the implementation science community.

## Limitations

This study had several limitations. First, the evaluation team chose not to use the full CFIR analysis approach which involves rating of CFIR constructs by case [12]; although this approach offers the ability to compare across sites, the 4 COIs on which this evaluation was based is a small number of sites and therefore the application was not appropriate. Second, similar to the analysis conducted by Damschroder 2013 [12] for their evaluation, discrepancies in coding were not quantified in this study. However, no issues were encountered while reaching consensus on disparate codes [12], which suggests that use of the constructs as a priori codes in a structured analytical tool is appropriate. Finally, this study may not highlight the potential application of use of CFIR in other healthcare contexts and additional studies may be needed.

## Abbreviations

COI: Center of Innovation
CFIR: Consolidate Framework for Implementation Research
CIH: Complimentary and Integrative Health
OPCC&CT: Office of Patient-Centered Care and Cultural Transformation
PCMH: patient-centered medical home
PCC: patient-centered care
PR: process redesign
VA: Department of Veterans Affairs
